# Fracture damage and energy dissipation of granite based on continuous-discontinuous coupling

**DOI:** 10.1371/journal.pone.0322377

**Published:** 2025-05-07

**Authors:** Run-Zhi Jia, Hong-Jie Duan, Meng-Zhen Su, Xiao-Zhi Liu, Yun-Juan Chen

**Affiliations:** 1 China National Chemical Communications Construction Group Co., Ltd., Jinan, China; 2 Shandong Highway Engineering Technology Research Center Co., Ltd., Jinan, China; 3 School of Civil Engineering, Shandong Jianzhu University, Jinan, China; China University of Mining and Technology, CHINA

## Abstract

A three-dimensional continuous-discontinuous numerical model was established through FLAC3D-PFC3D simulation coupling to study the fracture damage and energy dissipation characteristics of granite under varying confining pressures, based on X-ray diffraction and laboratory mechanical tests. The results demonstrated strong agreement between the simulation outcomes of the coupling model and the laboratory experiments, particularly concerning mineral composition. At approximately 65% of the peak strength of granite, internal cracks emerged and propagated rapidly around particles with weaker strength, resulting in the formation of primary “X” type cracks that diagonally penetrated the rock sample, accompanied by secondary tensile cracks. As the confining pressure increased, the ratio of tensile cracks in rock sample failure decreased, while the ratio of shear cracks increased. This transition from brittle to plastic failure in granite was effectively inhibited by confining pressure, which altered the failure process. Interestingly, granite exhibited a consistent energy evolution pattern under varying confining pressures. Prior to reaching peak strength, energy accumulation predominated, shifting to dissipation and release after the peak was surpassed. Notably, the impact of confining pressure on fthe elastic deformation energy of the rock mass was more significant than on dissipative deformation energy.

## Introduction

Throughout the extensive and intricate geological tectonic sequences, the natural rock formations have resulted in numerous primary cracks and damage flaws. Simultaneously, the development of subterranean rock structures alters not only the stress conditions of the adjacent rock but also perpetuates the detailed damage within the rock, leading to changes in its physical and mechanical properties. In the field of underground rock engineering, granite, a frequently encountered surrounding rock, holds immense importance for examining the unevenness in its mineral makeup and mechanical characteristics in relation to the rupture damage and the energy loss principles of the adjacent rock.

In the realm of rock mechanics research, indoor testing stands as the fundamental and most efficient technique [1]. Numerous researchers, both domestically and internationally, have conducted extensive experimental research on the extensive and minute rupture damage and the laws governing energy loss in rock formations, encompassing various tests like indoor mechanical examinations [2–4], electron microscope scans [5–7], acoustic emission assessments [5–11], among others. Lately, the development of numerical simulation and modeling methods has become crucial for examining the laws governing rupture damage, energy buildup, and dissipation in rock formations. Certain scholars, through the integration of indoor mechanical assessments and computational simulation techniques, examined the complete sequence of crack formation, spreading, and infiltration in rocks with distinctly contoured fractures [12]. The expansion of micro-cracks inside the rock mass and the variation in rock mass damage were analyzed using numerical simulation methods [13]. Some scholars have explored the dynamic tensile mechanical behavior of granite at the mineral particle scale and proposed a new three-dimensional equivalent crystalline model. They studied and discussed the macroscopic and microscopic fracture damage processes of the samples, as well as the impact of the crystal-to-unit size ratio on the dynamic tensile mechanical behavior of granite [14]. For another example, through indoor direct shear tests and the PFC2D discrete element program, the macroscopic and microscopic shear mechanical behavior of a through-type saw tooth rock structure surface was systematically studied [15]. By establishing a macroscopic and microscopic composite damage model of the rock mass, the development and changes of micro-cracks within the rock mass, as well as the macroscopic deformation characteristics, were analyzed from both macroscopic and microscopic perspectives [16]. Alternatively, using a particle flow discrete element program, a true tri-axial numerical model of the rock mass was established to study the effects of intermediate principal stress on the deformation, strength, damage, and failure characteristics of the rock mass under true tri-axial stress conditions [17]. Some scholars have used the particle flow simulation software PFC2D to analyze the energy and micro-crack evolution patterns during the failure process of red sandstone [18]. Others have studied the crack propagation and energy evolution of pre-fabricated joint sandstone under tri-axial compression, finding that the peak values of bond energy and strain energy in the samples are directly proportional to the pre-fabricated joint angle, while the peak strain of bond strength and peak strain energy are inversely proportional to it [19].

Previous studies on the damage and energy dissipation laws of granite rupture have primarily focused on internal cracks within the rock body, neglecting the external stress state of the surrounding rock. This paper aims to establish a correlation between the mineral composition properties and macroscopic rupture of the rock body by incorporating both macroscopic and microscopic analyses. Additionally, continuous-discontinuous coupled granite specimens will be used to investigate the rupture process under varying enclosing pressures, thereby examining the damage and energy dissipation laws.

## Materials and methods

### Continuous-discontinuous coupling modeling and parameter calibration

#### Granite mineral composition and compression rupture test.

**Fig 1:**
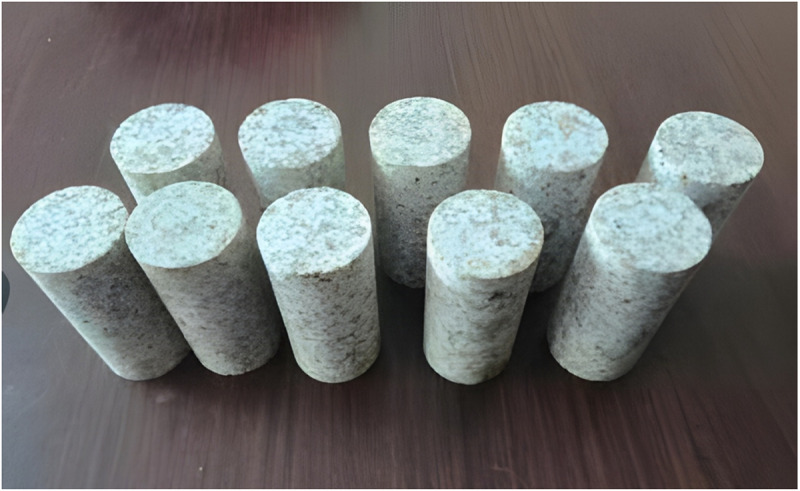
Granite sample.

The granite rock samples used in the experiment exhibited a silver-white hue, possessed a uniform texture, and displayed no discernible joints. In accordance with the guidelines set forth by both the International Rock Mechanics Society (IRSM) and China’s regulations pertaining to rock mechanics testing [20], cylindrical standard specimens were fabricated from the collected rock samples, with a diameter of 50 ± 0.3 mm and a height of 100 ± 0.3 mm. The end face of each specimen was required to be perpendicular to its axis, with any deviation strictly controlled within 0.25°. Additionally, the unevenness of the end face was limited to 0.05 mm, as depicted in Fig 1. The average mass of these standardized specimens amounted to approximately 505 g, while their average density measured at around 2,573 kg/m^3^; moreover, an average porosity level of approximately 2‰ was observed.

**Fig 2:**
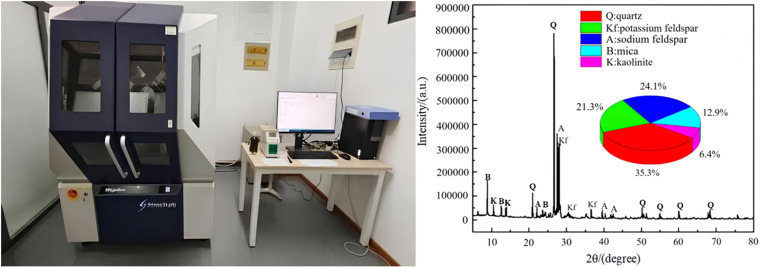
X-ray diffractometer and mineral composition.

In order to determine the mineral composition of the granite rock samples from a microscopic perspective, XRD diffraction tests were conducted on the specimens shown in Fig 1. The Smartlab SE, an intelligent multifunctional X-ray diffractometer from Shandong University of Architecture, was used for the XRD analysis by placing finely ground granite powder into the instrument. The obtained test data were then semi-quantitatively and physically analyzed using Jade software to derive the following mineral compositions of granite: quartz (35.3%), potassium feldspar (21.3%), sodium feldspar (24.1%), mica (12.9%), and kaolinite (6.4%) by mass ratio. These results are presented in Fig 2 along with details about the testing equipment.

**Fig 3:**
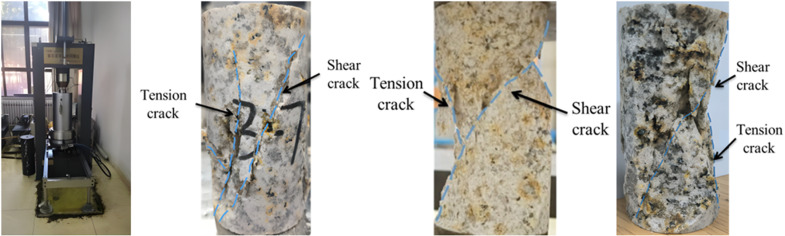
TAW-1000D testing machine and specimen fracture morphology.

According to the results of the XRD diffraction test for mineral determination, three groups of granite specimens with better homogeneity were selected for uniaxial compression testing. The TAW-1000D rock rheology disturbance tester was used as the testing instrument, applying a force at a rate of 10 N/s initially, and then switching to displacement loading at a rate of 0.1 mm/min after complete contact was made. Fig 3 illustrates the test equipment and rupture morphology of the three groups of granite specimens.

According to the basic principle of the Mohr-Coulomb failure criterion and the analysis of the sample fracture morphology in Fig 3, it can be seen that after uniaxial compression of the three groups of samples, a shear main fracture plane running through the upper and lower end faces of the entire sample is formed, and a certain tensile failure plane is derived near the main fracture plane. The macroscopic rupture morphology is primarily dominated by shear rupture, with some contribution from tensile rupture. The main rupture surface appears relatively smooth in the damaged section, resulting in fewer attached flocculent crystal particles. Since the main fracture plane is the shear plane that runs through the upper and lower end faces of the entire sample, the tension fracture plane primarily appears on the surface of the sample. The sample in the figure is spliced after the test, and some crushed rock blocks are difficult to splice. It can be observed that the shear main fracture plane is a cross-section, and the tension failure plane is rough and uneven after the test.

#### FLAC3D-PFC3D coupling model and parameters.

**Fig 4:**
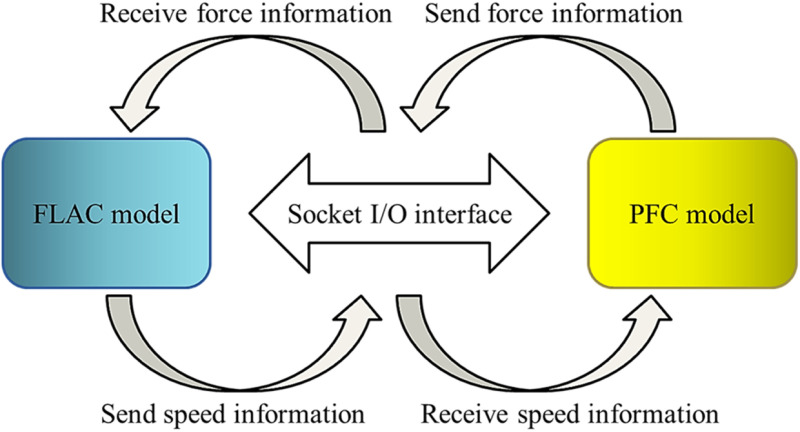
FLAC-PFC coupling calculation principle.

FLAC3D, a software for continuous deformation finite difference analysis, enables macroscopic analysis of the mechanical behavior of rock bodies. However, it may halt calculations when the simulated unit experiences excessive deformation due to the inability to solve the unit stiffness matrix. On the other hand, PFC3D is a software for discontinuous deformation discrete element analysis that allows a micro-perspective analysis of rupture and damage characteristics using granular units. The calculation method of the discrete element is based on discrete particle units, which can be separated from each other. The discrete element program updates the motion equations between particle units by continuously judging the contacts. Although this program may also encounter calculation issues due to excessive granular unit deformations and computational inefficiency caused by numerous contact judgments, coupling these two software programs can achieve dual optimization in both computational efficiency and accuracy. The Socket I/O interface facilitates data transmission and exchange between different domains at the contact boundary between continuous and discrete domains (as shown in Fig 4). In this study, we establish a continuous-discontinuous coupling model using an interface coupling method [21]: setting the PFC3D model component wall (Wall) on the surface of FLAC3D model elements (unit Zone or structural unit), which serves as an exchange medium for coupling variables between FLAC3D and PFC3D.

**Fig 5:**
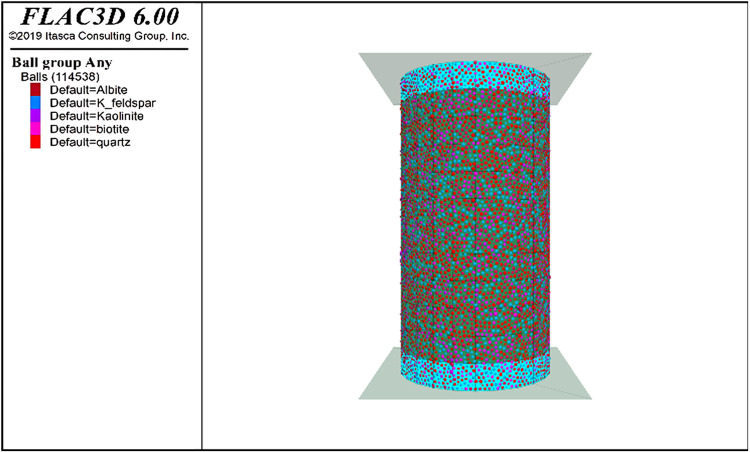
Continuous-discontinuous coupling model.

The process of continuous-discontinuous modeling in this paper is as follows: In the first step, the PFC3D module was loaded into FLAC3D software, and the shell element in FLAC3D was used to build a film cylinder with a diameter of 50 mm and a height of 100 mm. The “zone-wall” command was used to generate the film cylinder into a coupled wall grid, and then the loading plate of the model is created. During the subsequent phase, PFC particles were produced within the film cylinder, leading to the random categorization of granite’s various mineral compositions. Initial computations were conducted to verify the absence of any congruence among these mineral particles. Subsequently, fundamental characteristics like porosity, density, and the internal friction angle of the individual meta-particles were determined based on elementary physical parameters derived from indoor experiments, as depicted in Table 1 Simultaneously, given granite’s robustness, dense nature, vulnerability to brittle damage, and complex mineral makeup, the linear parallel bonding model was selected for the intrinsic model in the discrete element program [22]. At the third stage, the spherical system achieves equilibrium through particle friction, and the additional energy from the second phase is eliminated to develop the initial equilibrium model. In the fourth step, the continuum-discontinuum coupling model can be obtained by grouping the mineral particles according to the results of the X-ray diffraction test, as shown in Fig 5.

**Table 1:** Basic parameters of model.

Particle radius/mm	Poriness/%	Density/(kg·m^-3^)	Internal friction angle of parallel bonding/°
0.75×10^–3^	0.2	2 573	0.5

**Fig 6:**
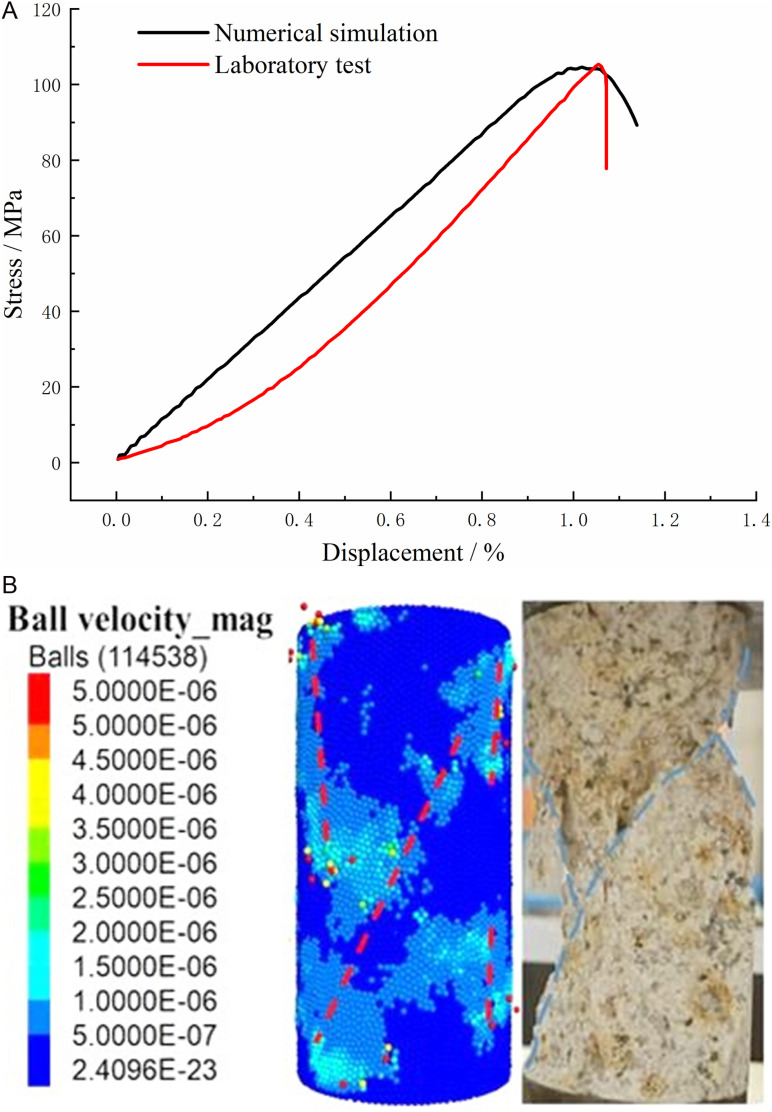
Comparative analysis of numerical simulation and laboratory test.

Indoor mechanical experiments yielded mechanical metrics like elasticity modulus, internal friction angle, and granite’s Poisson’s ratio of granite. Conversely, the standard bond strength ($\sigma_{n}$), shear bond strength ($\tau_{n}$), stiffness ratios of normal and shear ($k_{n}^{*}/k_{s}^{*}$), particles’ effective contact modulus ($E^{*}$), parallel adhesion’s contact modulus ($\overset{―}{E^{*}}$), stiffness ratios of normal to shear adhesion among individual elemental particles ($\overset{―}{k_{n}^{*}}/\overset{―}{k_{s}^{*}}$), and the internal friction angle of parallel bonding (f) were not derivable through indoor testing as per the pertinent literature [23–26]. The trial-and-error approach replaces experimental real function variables with the functional variables of current fine parameters to derive the values of each fine parameter among discrete elemental particles, subsequently refining them to develop a three-dimensional coupled granite numerical model. Table 2 displays the detailed parameters of the view. The coupling model depicted in Fig 5 undergoes parameterization and compression simulation, with a comparative analysis of numerical simulations and indoor test outcomes displayed in Fig 6.

**Table 2:** Table of mesoscopic parameters of granite numerical model.

Mineral name	Contact modulus /GPa	Standard bond strength /MPa	Shear bond strength /MPa	Stiffness ratio	Density /(kg·m^-3^)	Internal friction angle of parallel bonding /°	Damping coefficient	Poriness /%
Mica	1.36	3.85	6.60	2.1	2 573	0.5	0.6	0.2
Potassium feldspar	3.94	9.63	16.50	2.1	2 573	0.5	0.6	0.2
Sodium feldspar	3.13	8.86	15.18	2.1	2 573	0.5	0.6	0.2
Quartz	5.17	13.86	25.08	2.1	2 573	0.5	0.6	0.2
Kaolinite	0.82	2.31	3.96	2.1	2 573	0.5	0.6	0.2

From Fig 6a, it is evident that both indoor testing and numerical simulation demonstrate a pronounced, hard, and brittle behavior in the stress-strain relationship of granite. The rock body quickly reaches peak strength after failure, with no distinct initial fissure compaction stage observed in the numerical simulation due to the absence of initial fissures. However, despite some deviation in values, there is a high degree of similarity between the two curves during the pre-peak elasticity stages. Furthermore, strong agreement exists in terms of peak intensity values between them, indicating that the calibration results of the continuous-discontinuous coupling model and parameters accurately capture the stress-strain relationship. In Fig 6b, considering the granite rupture morphology, both indoor testing and numerical simulation show diagonal shear rupture surfaces within the rock specimen. The concentrated range of particle flow displacement also aligns with this diagonal shear rupture surface observed in both cases, demonstrating good agreement between them. Additionally, several noticeable tensile fracture surfaces appear around the destructive shear rupture surface, which is the consistent with overall morphology observed from both approaches. Therefore, based on these consistent findings regarding stress-strain relationship and rock body rupture morphology, it can be concluded that utilizing this established continuum-discontinuity coupling model along with calibrated parameters provides an accurate framework for conducting subsequent research.

## Results and discussion

### Mechanical characteristics of granite rupture damage

#### Analysis of granite fine fracture damage process under no lateral limit condition.

**Fig 7:**
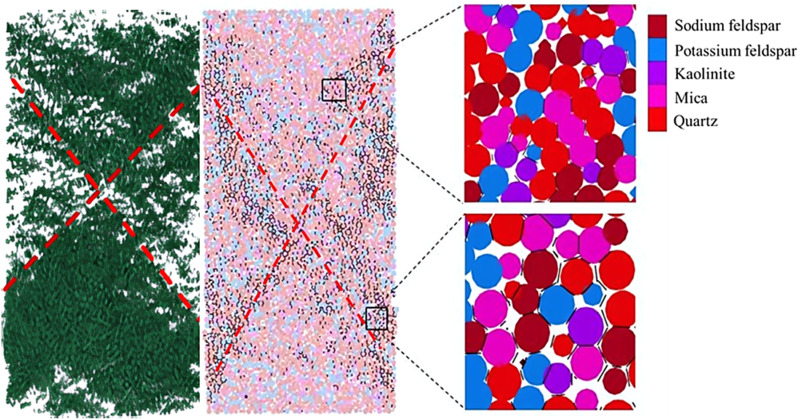
Granite bond failure characteristic map.

Under the action of external loads, internal microcracks are continuously generated and developed in the rock. As the rock body destabilizes and becomes damaged, these micro-cracks expand and eventually form a macroscopic fracture surface. Hence, the rupture and damage process of the rock body can be seen as the progression of internal micro-crack initiation, expansion, and penetration. In the numerical simulation test, there are no initial micro-cracks. Based on the characteristics of the PFC3D discrete element parallel bonding model, the discrete elemental particle units can establish disc-like adhesive contacts between them, providing a better representation of the micro-crack behavior among various mineral compositions during rock rupture. When the normal force or shear force between mineral particle units exceeds the corresponding normal bond or shear bond strength, the bond contact between discrete element particles will is destroyed, resulting in tension cracks or shear cracks. By programming the fish language in PFC3D to extract bonding failures between particles during the failure process, shear cracks and tensile cracks can be identified. Fig 7 illustrates the damage characteristics resulting from the uniaxial compression of granite, showcasing fine-scale rupture damage features.

The compression of granite generates oblique shear rupture surfaces through the specimen, as depicted in Fig 7 This observation aligns with the experimental findings, with the key distinction being that numerical simulations mitigate errors introduced during testing due to instrumentation limitations and human factors. Consequently, the numerical simulation exhibits two diagonal shear rupture surfaces through the specimen, forming an “X” distribution pattern. Furthermore, upon magnification of the destruction region in the simulation, observations reveal the rupture of internal mineral compositions. The small, short lines in the illustration symbolize cracks between mineral particles, primarily concentrated around mica and kaolinite minerals. This suggests that minerals like mica and kaolinite, characterized by lower hardness, are susceptible to weak points in the destruction of granite specimens.

#### Mechanical characterization of granite under different lateral limit conditions.

**Fig 8:**
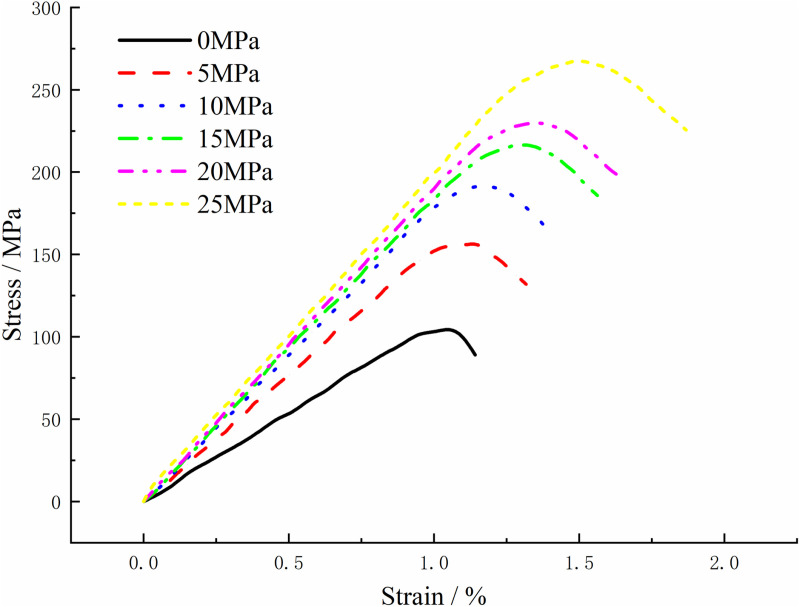
Stress-strain curves of granite under different confining conditions.

**Fig 9:**
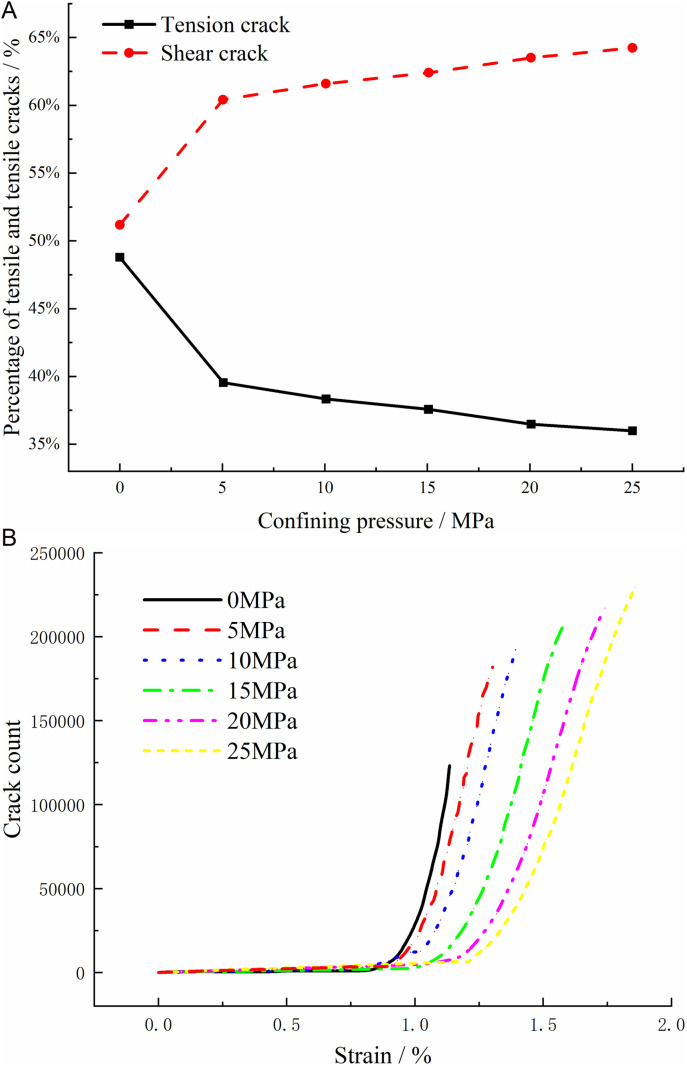
Relationship between granite crack propagation characteristics and confining pressure.

The deformation and damage analysis of granite under different side-limit conditions were conducted to investigate their influence on the mechanical characteristics. The “zone apply stress” command in FLAC3D is applied to the coupling wall to exert confining pressure. At the same time, the “wall” contact attribute in PFC3D is defined to implement the confinement pressure control. The coupling module “coupling interface update” of FLAC3D-PFC3D is activated to ensure that the stress state of FLAC3D is updated in real time with the particle state of PFC3D, achieving confining pressure application. This study is based on a continuous-discontinuous coupled numerical model. Six working conditions were considered for the side-limit perimeter pressures: 0 MPa, 5 MPa, 10 MPa, 15 MPa, 20 MPa, and 25 MPa. The stress-strain curves of granite under these various side-limit conditions are displayed in Fig 8, while the expansion and cracking features can be observed in Fig 9.

With the increase in peripheral pressure, as shown in Fig 8, the stress-strain curve of the granite specimen shows a trend of steepening. Consequently, the elastic modulus of the rock body increases, accompanied by a significant rise in peak strength. Notably, at low peripheral pressure levels, the granite peak undergoes rapid instability and decline following the destruction of the rock body, reflecting brittle failure. Conversely, at high peripheral pressure levels, the post-destructive behavior of the rock body reveals a more pronounced plastic state in the granite. It is observable that as the peripheral pressure increases, the plastic state of the rock body becomes more pronounced. This implies that intensifying the perimeter pressure increases the granite’s resistance to damage, causing it to gradually transition from brittle failure to plastic deformation.

Based on the characteristics of PFC3D discrete element parallel bonding model, bonding contact exists between discrete element particles, and this bond is maintained until the normal or shear stress between the particles exceeds their respective bonding strength thresholds. When the normal stress surpasses the normal bonding strength or the shear stress exceeds the shear bonding strength, the bonding contact is disrupted, leading to the formation of tensile cracks or shear cracks. To analyze this process, a command can be implemented to detect and quantify the number of cracks. This approach enables the identification of bonding failures between particles during the model’s failure process, allowing for the differentiation between tensile cracks and shear cracks. The increase in perimeter pressure leads to a higher proportion of shear cracks and a lower proportion of tensile cracks within the granite, as shown in Fig 9a. This shift explains the susceptibility to splitting damage on the cavern surface during excavation. When the perimeter pressure is released, inducing tensile damage in the cavern wall is more likely, as the perimeter pressure within the wall is reduced to zero. Conversely, when the perimeter pressure effectively constrains the sides, the granite mainly experiences shear damage. Analysis of Fig 9b reveals that the number of cracks propagating within the rock body is directly correlated with the perimeter pressure under various side-limiting conditions in granite. Additionally, the increase in perimeter pressure delays the rapid and unstable stages of crack propagation. Furthermore, when comparing rock bodies under the same strain, higher perimeter pressure results in fewer cracks, suggesting that the presence of perimeter pressure effectively limits or impedes the progression of granite damage. Notably, higher perimeter pressure boosts the loading capacity of granite, enabling greater deformation potential before damage occurs.

### Characterization of the energetic evolution of granites

In nature, the rock body exchanges energy with its external environment. When external forces from the outside world perform work on the rock body, mechanical energy $U_{0}$ is introduced into the system. This energy is partitioned into two forms: reversible elastic deformation energy $U_{0}^{e}$, which can be recovered upon unloading; and dissipative deformation energy $U_{0}^{D}$, which is irreversibly lost through processes such as plastic deformation, fissure expansion, and strength degradation. The energy relationship of the rock body can be described by the following equation [26]:


$$U_{0}=U_{0}^{e}+U_{0}^{D}$$ (1)

The total and elastic energy of each part of the rock mass unit in the principal stress space can be expressed as:


$$U_{0}=\int_{0}^{\varepsilon_{1}}\sigma_{1}d\varepsilon_{1}+\int_{0}^{\varepsilon_{2}}\sigma_{2}d\varepsilon_{2}+\int_{0}^{\varepsilon_{3}}\sigma_{3}d\varepsilon_{3}$$ (2)

$$U_{0}^{e}=\frac{\sigma_{1}\varepsilon_{1}+\sigma_{2}\varepsilon_{2}+\sigma_{3}\varepsilon_{3}}{2}$$ (3)

$$E_{i}^{e}=\frac{\sigma_{i}-\nu_{i}\left(\sigma_{j}+\sigma_{k}\right)}{E_{i}}$$ (4)

where $U_{0}$ is the total energy; $U_{0}^{e}$ is the elastic deformation energy; $U_{0}^{D}$ is the dissipative deformation energy; $\sigma_{i}$, $\sigma_{j}$ and $\sigma_{k}$ (where *i*, *j*, and *k* = 1, 2, and 3) are the first, second, and third principal stresses, respectively; $\varepsilon_{i}$ and $\varepsilon_{i}^{e}$ (*i* = 1, 2, and 3) are the total and elastic strains in the directions of the principal stresses, respectively; and $\nu_{i}$ and $E_{i}$ are Poisson’s ratio and elastic modulus.

**Fig 10:**
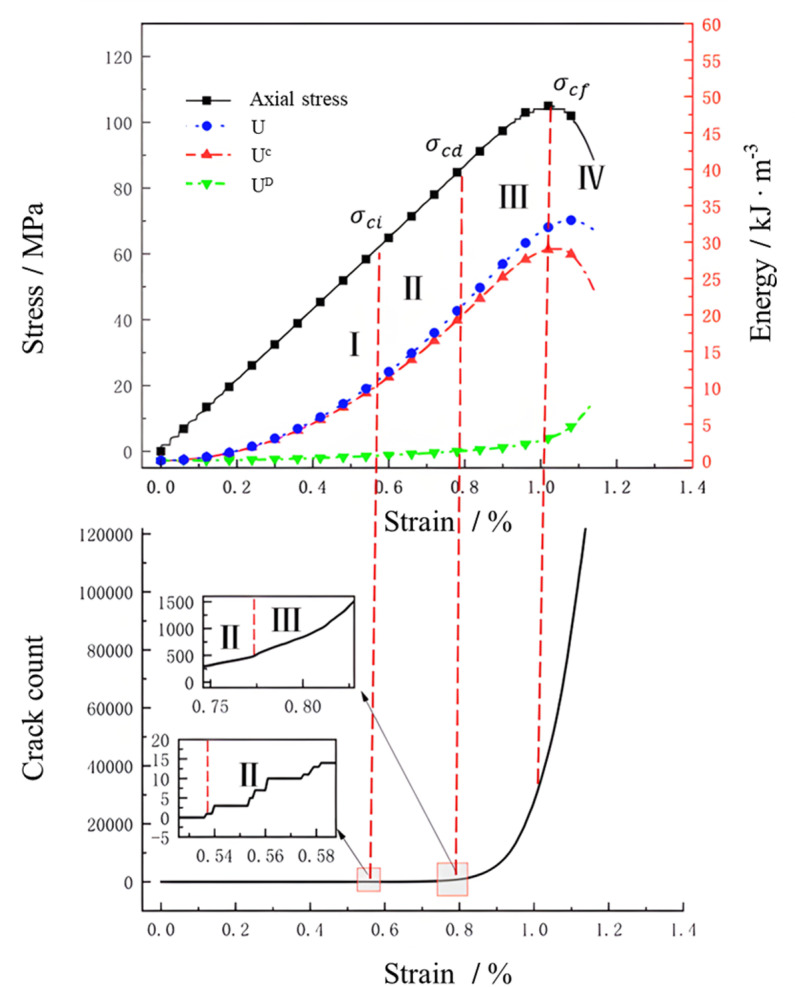
Energy evolution characteristics of granite.

The coupled numerical computation of FLAC3D-PFC3D was used to analyze the evolution of energy dissipation within the rock body, focusing on the uniaxial compression test. Through the use of the FISH language, the research extracted variations in stress, energy density, and the number of crack with strain during the compression process, as illustrated in Fig 10. Notably, U represents the total energy density introduced from external sources, $U_{e}$ stands for elastic deformation energy density, and $U_{D}$ refers to dissipative deformation energy density. It is important to note that the numerical simulation does not account for the initial microcracks and other defects in the rock body. Consequently, the analysis is limited to the four stages of granite behavior: elastic deformation, crack stable expansion, crack unstable expansion, and macroscopic damage. Key stress points in each stage include crack initiation stress ($\sigma_{ci}$), crack unstable expansion stress ($\sigma_{cd}$), and peak strength stress ($\sigma_{cf}$). Furthermore, the energy values corresponding to these characteristic stresses are identified as characteristic energy value points.

In the elastic deformation stage (Ⅰ), the rock body undergoes primarily elastic deformation, with no new cracks forming internally, as depicted in Fig 10. The work done by the external load aligns closely with the elastic deformation energy accumulated in the rock body, leading to minimal energy dissipation. As the axial strain increases, the rate of accumulation of elastic deformation energy gradually rises, with the work of the external force primarily transforming into internal elastic deformation energy. The release of this energy occurs when the external load is removed. The appearance of cracks within the rock body signifies the specimen’s transition from elasticity to the stable crack expansion stage. At this juncture, the stress corresponds to the cracking stress, denoted as $\sigma_{ci}$. This stage marks a shift in the behavior of the rock sample from elastic deformation to crack propagation.

During the crack stable expansion stage (II), the initial crack within the rock body starts to gradually and steadily expand as the stress reaches the crack initiation stress $\sigma_{ci}$. Accordingly, the growth of elastic strain energy gradually diminishes. Simultaneously, the work done by the external force on the rock body is partially converted into elastic deformation energy. Furthermore, a portion of the energy dissipates as friction during the crack expansion process.

During the stage of unstable crack expansion (Ⅲ), plastic deformation occurs within the rock body, leading to discernible nonlinear changes in the stress-strain curve. This phase is characterized by a significant decrease in the accumulation rate of elastic deformation energy within the rock mass. Consequently, the curve gradually veers away from the total energy input from external sources. Correspondingly, the stress experienced at this point is represented by the stress from the unstable crack’s expansion ($\sigma_{cd}$). Moreover, the dissipation rate of deformation energy within the rock mass gradually increases, accompanied by the rapid accumulation of internal cracks. The external force exerted on the rock mass deformation, does work, and propagates cracks within the rock in the forms of friction, plastic deformation, and crack propagation.

When the stress in the rock body reaches the peak strength stress $\sigma_{cf}$, it enters the macro-destruction stage (Ⅳ). At this stage, the internal accumulation of elastic deformation energy decreases rapidly, and the dissipation of deformation energy increases abruptly. Subsequently, the rock body initiates slipping along the macro-rupture surface, leading to its destruction. As the rock body progresses through the macro-destruction stage, the elastic deformation energy gradually declines from its peak value, while the dissipative deformation energy experiences a significant surge. The process continues with the extension and penetration of cracks until the rock body ultimately reaches complete destruction, releasing the accumulated energy within.

**Fig 11:**
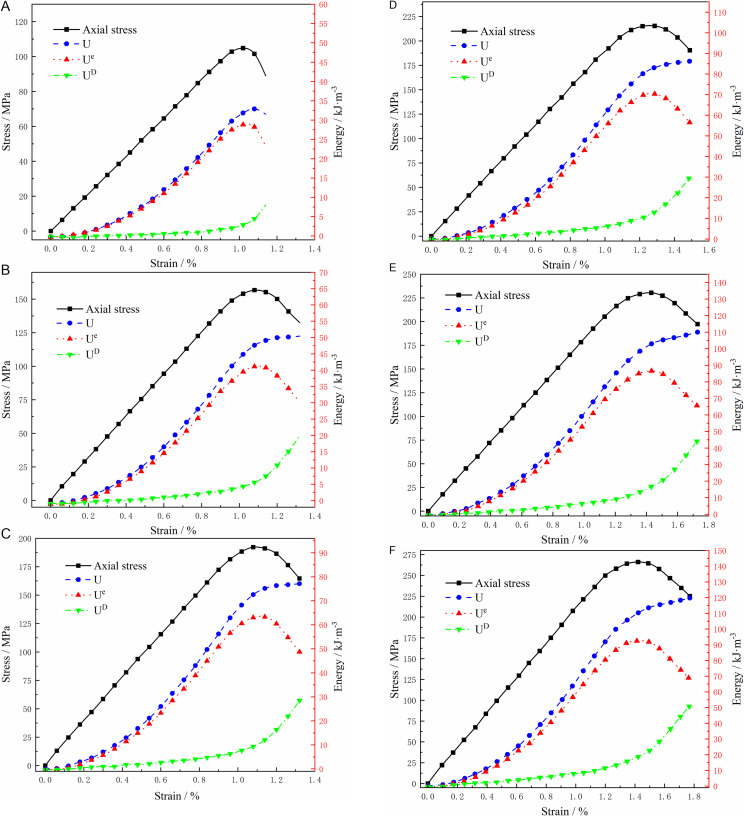
Energy evolution curve of granite under different confining pressures.

The FISH language was used to compile the energy evolution curves of elastic deformation energy, dissipative deformation energy, and total energy with axial strain in granite under varying confining pressures. To explore the relationship between different types of deformation energy densities and the stress-strain process, the energy evolution laws were plotted alongside the stress-strain curves in a single graph for comparative analysis, as illustrated in Fig 11.

The energy accumulation and dissipation law of the granite compression process under different pressures is similar, as shown in Fig 11. Initially, in the elastic deformation stage, the external energy input is primarily in the form of elastic deformation energy stored within the rock body, constituting over 90% of the total energy. This energy predominantly accumulates within the granite and can be fully released upon unloading at this stage. As the compression progresses into the stable crack expansion and unstable expansion stages, cracks begin to develop, leading to plastic deformation. Consequently, the accumulation rate of elastic deformation energy decreases, while the dissipation rate increases. Notably, the rate of energy dissipation rises significantly with higher peripheral pressures. Before reaching peak strength, the granite undergoes energy accumulation, with the storage density of elastic energy increasing alongside the rising peripheral pressure. Following the peak strength, the total energy growth decelerates, accompanied by a reduction in elastic deformation energy and an increase in dissipative deformation energy. Importantly, the post-peak decline in elastic deformation energy slows as peripheral pressure increases, leading to a substantial release of dissipative deformation energy during the destruction of the rock body. This process results in the amplification of peak and residual strengths, primarily driven by the dissipation and release of energy during this stage.

According to the energy evolution curve of granite in Fig 11, the energy density values for granite’s crack initiation stress ($\sigma_{ci}$), crack instability extension stress ($\sigma_{cd}$), and peak strength stress ($\sigma_{cf}$) were extracted at various enclosure pressures, as presented in Table 3.

**Table 3:** Characteristic energy density values of granite under different confining pressures.

Confining pressure/MPa	Peak strength/MPa	$U_{ci}^{e}$	$U_{cd}^{e}$	$U_{cf}^{e}$	$U_{ci}^{D}$	$U_{cd}^{D}$	$U_{cf}^{D}$
0	105	4.2	20.8	28.9	0.37	1.4	2.9
5	157	3.8	30.2	41.5	0.6	3.4	7.4
10	192	4.9	46.8	63.1	0.9	5.0	9.8
15	218	5.7	47.1	70.6	1.0	5.1	12.4
20	233	5.9	61.3	86.4	1.1	7.2	15.7
25	267	6.2	63.9	92.1	1.7	8.7	17.0

**Fig 12:**
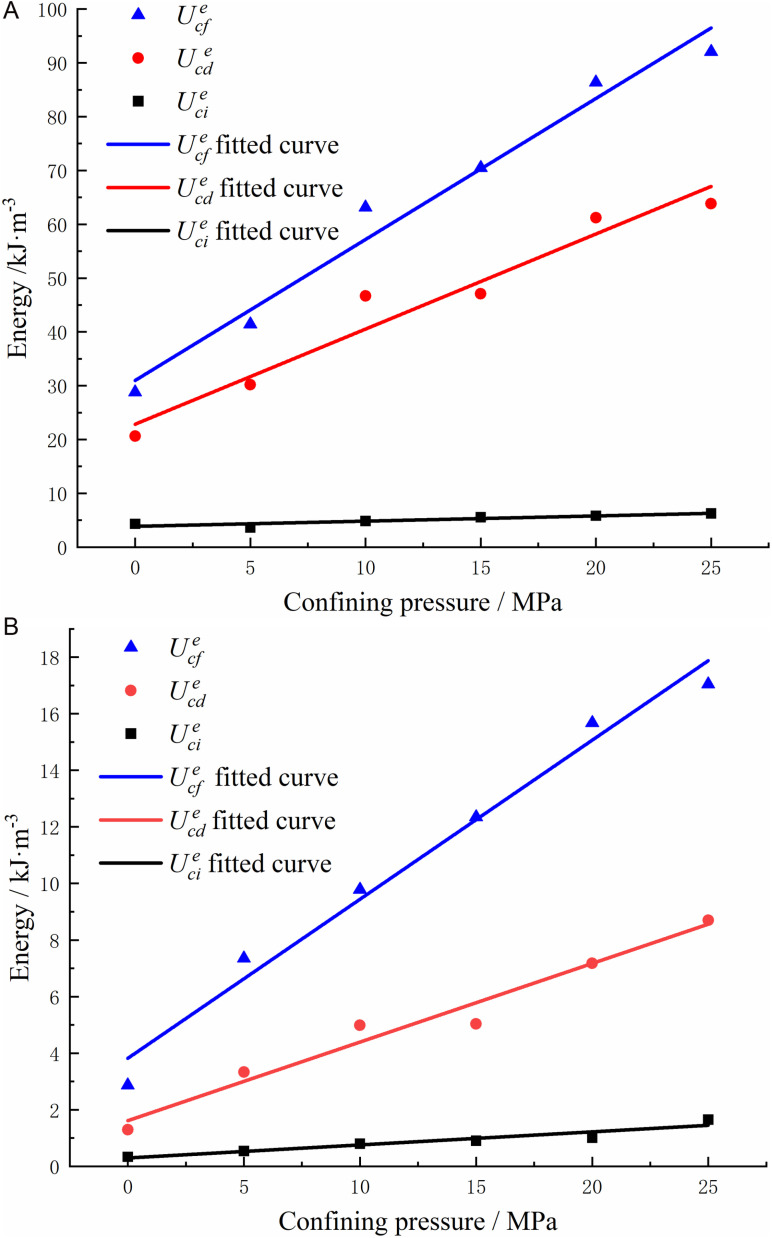
Relationship between energy density eigenvalue and confining pressure.

The eigendensity values of elastic deformation energy, represented as $U_{ci}^{e}$, $U_{cd}^{e}$, and $U_{cf}^{e}$, were extracted and fitted. Similarly, the eigendensity values of dissipative deformation energy, denoted as $U_{ci}^{D}$, $U_{cd}^{D}$, and $U_{cf}^{D}$, were also obtained and fitted. Fig 12 illustrates the relationship between each energy density eigenvalue and the enclosing pressure.

The relationship between the eigenvalues of each energy density and the perimeter pressure is evident in Fig 12, demonstrating a positive proportional correlation. By analyzing the slope of the energy eigenvalues, it becomes apparent that the perimeter pressure has a more pronounced impact on the elastic deformation energy of granite compared to the dissipative deformation energy. This observation suggests that as the perimeter pressure increases, a greater amount of energy from external sources is stored as elastic deformation energy within the rock body. Consequently, when the accumulated elastic deformation energy surpasses its threshold, the rock body undergoes damage, leading to the release of stored energy. Consequently, the rock body will be damaged and release energy when the accumulation of elastic deformation energy exceeds its limit.

## Conclusion

In this paper, based on the XRD analysis of the granite mineral composition, a three-dimensional continuous-discontinuous coupled numerical model of granite was established to study the macro and microfracture damage and energy evolution characteristics of granite under different confining pressures. The conclusions are as follows:

(1) The research method, involving the continuous-discontinuous coupling numerical method, aligns closely with the test results, thus demonstrating its feasibility. As the load approaches approximately 65% of the peak strength of granite, initial cracks emerge within the rock mass. These cracks then progress and widen in areas with weak mineral compositions, such as kaolinite and mica. Over time, these cracks evolve into “X”-shaped shear fractures that traverse the rock samples diagonally, eventually giving rise to secondary tensile cracks.
(2) As the peripheral pressure increases, the proportion of tensile cracks gradually decreases, while the proportion of shear cracks gradually increases in the damage form of granite, leading to an increase in the number of bond damages inside the rock body. The unstable development stage of rock cracks is delayed as peripheral pressure increase. Furthermore, the presence of peripheral pressure inhibits the damage process of granite, transitioning it from brittle failure to plastic deformation.
(3) Before peak strength, granite is dominated by energy accumulation, and the elastic energy storage density increases with increasing peripheral pressure. After reaching peak strength, granite shifts its focus to energy dissipation and release. The increase in peripheral pressure results in a slower decline in the elastic deformation energy after the peak. This demonstrates a positive relationship between the dissipation of deformation energy, peak strength, residual strength, and peripheral pressure. It is worth noting that the effect of peripheral pressure on the elastic deformation energy of granite exceeds its effect on the dissipative deformation energy.

## Supporting information

S1 Data Drawing test data. (XLSX)
